# Polycystic Kidney Disease in the Medaka (*Oryzias latipes*) pc Mutant Caused by a Mutation in the Gli-Similar3 (glis3) Gene

**DOI:** 10.1371/journal.pone.0006299

**Published:** 2009-07-17

**Authors:** Hisashi Hashimoto, Rieko Miyamoto, Naoki Watanabe, Dai Shiba, Kenjiro Ozato, Chikako Inoue, Yuko Kubo, Akihiko Koga, Tomoko Jindo, Takanori Narita, Kiyoshi Naruse, Kazuko Ohishi, Keiko Nogata, Tadasu Shin-I, Shuichi Asakawa, Nobuyoshi Shimizu, Tomotsune Miyamoto, Toshio Mochizuki, Takahiko Yokoyama, Hiroshi Hori, Hiroyuki Takeda, Yuji Kohara, Yuko Wakamatsu

**Affiliations:** 1 Bioscience and Biotechnology Center, Nagoya University, Nagoya, Japan; 2 Graduate School of Science, Nagoya University, Nagoya, Japan; 3 Department of Anatomy and Developmental Biology, Kyoto Prefectural University of Medicine, Kyoto, Japan; 4 Graduate School of Science, The University of Tokyo, Tokyo, Japan; 5 Center for Genetic Resource Information, National Institute of Genetics, Mishima, Japan; 6 Department of Molecular Biology, Keio University School of Medicine, Tokyo, Japan; 7 Second Department of Medicine, Hokkaido University Graduate School of Medicine, Sapporo, Japan; University of Pennsylvania School of Medicine, United States of America

## Abstract

Polycystic kidney disease (PKD) is a common hereditary disease in humans. Recent studies have shown an increasing number of ciliary genes that are involved in the pathogenesis of PKD. In this study, the Gli-similar3 (*glis3*) gene was identified as the causal gene of the medaka pc mutant, a model of PKD. In the pc mutant, a transposon was found to be inserted into the fourth intron of the *pc/glis3* gene, causing aberrant splicing of the *pc/glis3* mRNA and thus a putatively truncated protein with a defective zinc finger domain. *pc/glis3* mRNA is expressed in the epithelial cells of the renal tubules and ducts of the pronephros and mesonephros, and also in the pancreas. Antisense oligonucleotide-mediated knockdown of *pc/glis3* resulted in cyst formation in the pronephric tubules of medaka fry. Although three other *glis* family members, *glis1a*, *glis1b* and *glis2*, were found in the medaka genome, none were expressed in the embryonic or larval kidney. In the pc mutant, the urine flow rate in the pronephros was significantly reduced, which was considered to be a direct cause of renal cyst formation. The cilia on the surface of the renal tubular epithelium were significantly shorter in the pc mutant than in wild-type, suggesting that shortened cilia resulted in a decrease in driving force and, in turn, a reduction in urine flow rate. Most importantly, EGFP-tagged pc/glis3 protein localized in primary cilia as well as in the nucleus when expressed in mouse renal epithelial cells, indicating a strong connection between pc/glis3 and ciliary function. Unlike human patients with *GLIS3* mutations, the medaka pc mutant shows none of the symptoms of a pancreatic phenotype, such as impaired insulin expression and/or diabetes, suggesting that the pc mutant may be suitable for use as a kidney-specific model for human *GLIS3* patients.

## Introduction

Polycystic kidney disease (PKD) is a common heritable kidney condition in humans. It is characterized by the appearance of fluid-filled cysts in the renal tubules and collecting ducts of the kidney, with pleiotropic lesions sometimes occurring in other organs, such as the liver, the retina and the pancreas (reviewed in [1]). Recent studies have proposed that renal cilia, which are immotile organelles projecting from the renal epithelium into the lumen of the nephric tubule or duct, play a crucial role in cyst formation. Animal models such as *Tg737^orpk^* and *Kif3a* mutant mice, which have atypically structured cilia in the renal tubule, have cystic kidneys (reviewed in [2]), suggesting that normally structured renal cilia are required for the maintenance of tubular lumen morphology. Mutations in *PKD1* and *PKD2*, which encode polycystin-1 and polycystin-2, respectively, are responsible for most cases of PKD in humans [3], [4]. Both polycystins localize in the primary cilia and evoke cellular responses to mechanostimuli of cilia, such as an increase in cytoplasmic Ca^2+^ [5], [6]. These findings imply that primary cilia bearing the polycystin-1/-2 complex function as mechanosensors of renal flow, sensing the rate of flow and thereby initiating the ciliary signal, which gives rise to the cellular response and the regulation of lumen size [7], [8]. Previous reports have suggested that PKD pathogenesis is associated with aberrant cell proliferation as well as altered planar cell polarity in the renal tubular and ductal epithelium [2], [9]–[12].

Studies of zebrafish and medaka PKD mutants have revealed that the ciliary beating motion is required on the surface of the renal epithelium for the generation of urine flow in the pronephric duct [13], [14]. In addition, in fish, a low urine flow rate is thought to lead to cyst formation. The role of the cilia in PKD pathogenesis differs in fish and mammals: the cilia in fish are motile and actively induce urine flow, whereas in mammals, the cilia merely serve as passive sensors of urine flow.

The medaka pc mutant is a piscine mesonephric model of PKD [15], meaning that the mutant develops typical PKD symptoms such as swollen renal tubules, fluid-filled cysts, and abdominal enlargement, all of which are inherited in an autosomal and recessive manner. The PKD phenotype in the pc mutant becomes histologically apparent soon after the completion of pronephric development (several days after hatching). As the mesonephros develops, the condition becomes more apparent, and, by the end of the animal's life, the abdomen has become severely distended. Although our previous findings revealed that pc mutants have cilia on the surface of the renal tubular epithelium, we did not determine whether these cilia are normal in terms of structure and motility [15]. While PKD patients and other animal models are known to have multiple disorders in which organs other than the kidney are affected, including retinal degeneration, hepatic fibrosis, *situs inversus*, and cystic disease in the pancreas or the liver [11], [12], medaka pc mutants do not develop notable pleiotropic lesions in addition to PKD. Given that PKD is a late-onset condition that progresses at a moderate rate in pc mutants, the medaka pc mutant has a relatively long life when compared with zebrafish pronephric models of cystic kidney, such as vHnf1, *double bubble*, and *oval* (*polaris/IFT88/osm-5*) mutants [16]–[23]. The combined features of the medaka pc mutant show that it is a good clinical model of the human form of the disease.

In this study, we identified Gli-similar 3 (*glis3*) as the causal gene of the pc mutant by positional cloning. Human *GLIS3* was recently identified as the gene responsible for a neonatal diabetes syndrome associated with congenital hypothyroidism, congenital glaucoma, hepatic fibrosis and polycystic kidneys [24]. Here we provide evidence that clearly indicates that a mutation in the *pc/glis3* gene causes PKD in medaka. Our data suggest that *pc/glis3* is involved in the renal ciliary function that is required for the production of urine flow and maintenance of the size of the renal tubular lumen.

## Materials and Methods

### Fish

The medaka pc mutant was first isolated by Dr. Hideo Tomita at Nagoya University [15] and has been maintained in a homozygous state over many generations (pc homozygotes develop into adults and are able to produce offspring). The pc homozygotes were crossed with the HNI-I strain [25], which is an inbred strain originating from a medaka population in northern Japan [26], and the resulting F1 generation was sib-mated to produce an F2 generation. A total of 847 F2 homozygotes were collected for locus mapping.

The developmental stages assigned to medaka are the same as those described elsewhere [27].

During experiments, medaka were handled in accordance with the Regulations for Animal Experiments in Nagoya University.

### Positional cloning

The linkage group within which the pc locus is located was determined using the M-marker system developed by Kimura et al. [28]. The BAC library constructed by Matsuda et al. [29] was used for chromosome walking. Four sequential BAC clones, 184A3, 198E6, 201K4, and 174E15 were isolated to span the area from the AU171175 marker to the pc locus. STS markers were identified from the end sequence of each BAC clone. More detailed information on the markers used is available on request.

### cDNA cloning of Glis family members and other genes

cDNAs were cloned by RT-PCR with primers designed on the basis of information in the medaka genome database (http://www.ensembl.org/Oryzias_latipes/) [30]. Cloned cDNA sequences have been deposited in the DDBJ database under the following accession numbers: *pc/glis3*, AB353137; *pc/glis3* short form, AB353138; *glis1a*, AB353139; *glis1b*, AB353140; *glis2*, AB353141. The *insulin* cDNA (AB257292) was isolated as described by Ogoshi et al. [31].

### In situ hybridization

A digoxigenin-labeled riboprobe was made from a cloned cDNA template using SP6 or T7 RNA polymerase after restriction enzyme digestion. In situ hybridization was performed as previously described [32]. To assess kidney staining, larvae were fixed by 4% Paraformaldehyde in PBS and the internal organs (gut, heart, liver, spleen, and reproductive organs), except for the kidneys, were removed through the abdomen before the previously described procedures.

### Histology

Paraffin sections were produced as previously described [33].

After *in situ* hybridization, the specimens were dehydrated in acetone, embedded in Technovit 8100 (Heraeus, Werheim, Germany) and sectioned. The cell nuclei were counterstained with 1% neutral red.

### Gene knockdown

Gene knockdown was performed by microinjection of antisense oligonucleotides into a one- or two-cell-stage embryo. The GripNA antisense oligonucleotides used in this study were *pc*-ATG-1 (5′-CACTCATGTCTAAAACGG-3′), *pc*-ATG-2 (5′-ACTAAACATGGACTGTGT-3′) and *pc*-SPD (5′-CAGATGTACCGAGCATTT-3′) (Active Motif, Tokyo, Japan). Each oligonucleotide was injected at a concentration of 1 ng per embryo.

### Fluorescent dye injection

Urine excretion assays were performed as previously described [13]. A 5% rhodamine-conjugated dextran (molecular weight: 10,000 Da; Molecular Probes) solution was injected into the common cardinal vein of 10-day postfertilization (dpf) fry anesthetized with 0.2 mg/ml tricaine (Sigma, Missouri, USA). Urine flow was observed under a stereoscopic microscope until the fluorescence reached the urinary bladder, or until the time following administration exceeded 30 min.

### Antibody staining and measurement of renal cilia length

The cilia of the hatchlings were subjected to length measurement. To avoid the segmental variation of the ciliary length, only the tubular segment of the pronephros was analyzed in comparison between the wild-type and the pc mutant. The samples were fixed in the same method as in situ hybridization. The primary cilia were visualized with anti-acetylated alpha-tubulin antibody (T6793, Sigma) and anti-rabbit IgG-FITC (81–6111; Zymed Laboratories, California, USA). Images of the cilia were obtained using a confocal fluorescence microscope (FV-1000, Olympus, Japan). Cilia length was determined by measuring at least 15 cilia for each individual using Image J 1.32j software (National Institute of Health). Only cilia that were clearly captured in fluorescence images and which had obvious ends (tip and root) were used for measurements.

### Detection of proliferating cells

Medaka embryos at 4 dpf (stage 30) and fry at 5-day posthatching (dph) were exposed to 10 mM BrdU (Roche Diagnostics, Basel, Switzerland) for 24 h. Embryos were dechorionated with hatching enzyme prior to exposure [34]. The embryos and fry were sliced into 5-µm sections after fixation in Bouin's solution. Cells that had incorporated BrdU were detected immunohistochemically with anti-BrdU mouse monoclonal antibody (Roche Diagnostics), anti-mouse IgG HRP and diaminobenzidine (DAB). The number of BrdU-positive cells was counted in each section taken from the whole kidney of 4 dpf embryos and in each of 15 sections taken from the most anterior region of the pronephros at 5 dph.

### Subcellular localization assay of the pc/glis3 protein

Full-length pc/glis3 cDNA was inserted into the BamHI site of the pCS3-MT vector [35], and EGFP cDNA was subcloned into the EcoRI/XhoI site to tag the C-terminus of the pc/glis3 protein. The resultant vector was transfected into a mouse renal epithelial cell line (Dai1 cells) [36] to examine the sub-cellular localization of the GFP-tagged pc/glis3 protein. Cells were cultured on glass coverslips coated with human collagen IV (50 mg/ml) at 33°C. Sub-confluent cells were incubated overnight in 1000 µl of culture medium to which 50 µl of serum-free medium containing 5 µl of Gene juice (Novagen, Darmstadt, Germany) and 0.5 µg of the cDNA expression construct was added per well. After primary cilia were formed, cells were fixed in ice-cold methanol/acetone [1∶1] for 10 min, permeabilized in 0.1% Triton X-100 for 20 min and quenched in PBS (137 mM NaCl, 2.6 mM KCl, 6.5 mM Na_2_HPO_4_,and 1.5 mM KH_2_PO_4_) containing 1% BSA for 1 h at room temperature. Cells were then incubated with mouse anti-acetylated alpha-tubulin antibody (clone 6-11B-1) [1∶2000] at room temperature for 2 h. Cells were washed in PBS and incubated with Alexa555-conjugated goat anti-mouse IgG for 1 h. Fluorescence was visualized with an IX70 microscope (Olympus, Tokyo, Japan). Digital images were processed by MetaMorph (Molecular Devices, Downingtown, PA, USA).

## Results

### The pc locus encodes a medaka ortholog of *GLIS3* (Gli-similar3)

**Figure 1:**
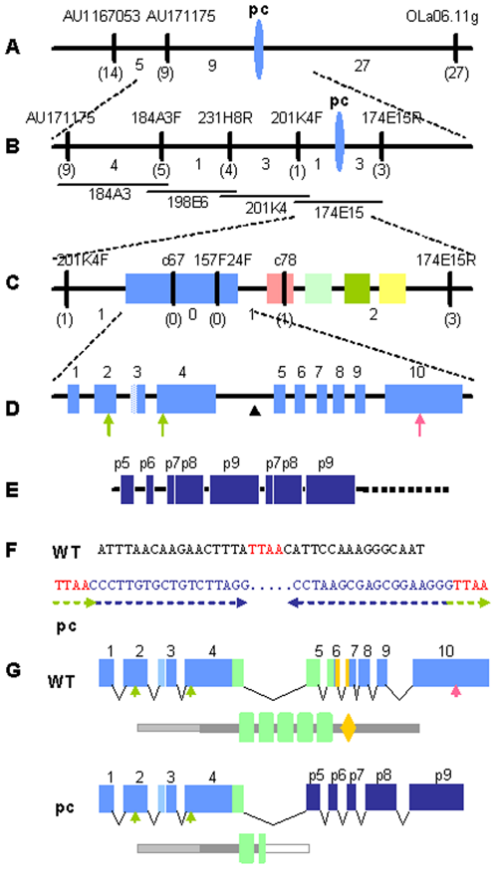
Positional cloning of the pc gene. (A) High-resolution recombination map around the pc locus on LG12. Numbers under the bar indicate the number of recombination events that occurred between neighboring markers. Numbers in parentheses show the number of recombinants found for the marker above the bar (among 1694 meiotic events). (B) BAC contig covering the pc locus. Physical mapping positioned the pc locus between the two markers 201K4F and 174E15R. The BAC MF001SSA174E15 contains the pc locus. (C) Distribution of genes predicted by Genscan. The 174E15 BAC insertion was sequenced using the shotgun method. Five genes, similar to *glis3* (blue), *RFX3* (pink), *SMAD4* (light green), *BMP10* (green) and *catenin arvcf-2ABC* (yellow), respectively, were predicted in this region. (D) Structure of the *pc/glis3* gene. The *pc/glis3* gene consists of 10 exons, with the third exon alternatively spliced to produce two different mRNAs with two different presumptive start codons. The positions of the start and the stop codons are indicated by arrows below the diagram of the gene structure. The arrowhead indicates the insertion point (5261/5726). (E) Structure of the insertion in the pc mutant. By comparing the sequences of the pc mutant cDNA and the pc genome, regions behaving as exons were identified for the pc locus. Three exonic sequences, p7, p8 and p9, were found at least twice in the pc mutant mRNA. In the pc genome, this fragment was found in the insertional region indicated by the arrowhead in (D). The sequence of the 3′ region of the insertion (dotted line) is unknown. (F) Sequence of the insertion point at the pc locus. A transposon-like sequence (>10 kb) was inserted into the fourth intron (3′ to the 5261 nucleotide of the 5726 bp intron). Like the insertion in the medaka rs-3 mutant, this insertion in the pc mutant contained 18 bp inverted repeats (blue) with 4 bp (TTAA in orange) duplications at the ends. Internal sequences of approximately 300 bp, with homology to the rs-3 transposon, were found adjacent to the 18 bp repeats at both ends (AB491224, AB491225). The arrowhead indicates the insertion point (WT) and the boundary of the transposon-like insertion and the innate sequence (pc). The other internal region of the insertion (>10 kb) is shown as a dotted line. (G) Predicted structures of WT and mutant pc mRNAs and proteins. The structures of mRNA (upper) and protein (lower) are shown for the WT and pc mutants. WT mRNA is transcribed from 10 exons. The third exon is alternatively spliced (light blue). The pc mutant mRNA lacks the 3′ region corresponding to exons 5–10. Instead, it has a different 3′ tail that is specific to the pc mutant (dark blue). The regions depicted in light green and orange indicate the sequences encoding the zinc fingers and the nuclear localization signal, respectively. The other exons are depicted in blue. Alternative splicing of exon 3 appeared to produce two distinct N-terminal domains, with the short form lacking the light-colored N-terminus. Zinc fingers are depicted as green boxes. The pc mutant protein lacks part of the second zinc finger motif and all three zinc fingers thereafter. The diamond-shaped region indicates a predicted nuclear localization signal. Other regions of the pc/glis3 protein are colored gray. The white box of the pc mutant protein corresponds to the amino acids produced from the pc insertional sequence.

In order to map the pc locus, we generated a total of 847 F2 siblings from crosses between pc mutants and an inbred strain with a different genetic background (HNI). First, by bulked segregation analysis using the M-marker system [28], we mapped the pc locus to linkage group 12 (LG12), before constructing a high-resolution recombination map around the pc locus using known polymorphic DNA markers on LG12. We found that the marker AU171175 was closest to the pc locus, with just nine recombination events (9/1694) occurring between them (Fig. 1A).

We then used BAC walking to construct a physical map with BAC clones that covered the pc locus. Precise recombination analysis revealed that BAC clone 174E15 contained the pc locus (Fig. 1B). This BAC clone was sequenced using the shotgun method [30].

Using the Genscan program, we found that BAC clone 174E15 contained five genes (Fig. 1C). Using additional recombination analysis using sequence-tag sites, we narrowed the pc locus region down to a 127 kb fragment containing only two genes, which were medaka orthologs of *GLIS3* and *RFX3*. We examined the mRNA expression of these two genes in the kidneys of wild-type and pc mutant medaka using RT-PCR. One of several primer sets for the *glis3* ortholog PCR-amplified a fragment in wild type but not in pc mutant kidney (Fig. S1A). Northern blot analysis using a fragment of *glis3* cDNA revealed *pc/glis3* mRNA in wild-type but not in pc mutant kidney (Fig. S1B), suggesting that *glis3* has a high likelihood of being the pc gene. We did not find any mutations in the RFX3 gene or cDNA (data not shown).

### *glis3* mRNA in pc mutants lacks part of the zinc-finger-encoding region

We isolated medaka pc cDNA from wild-type kidney by RACE PCR and determined the ORF sequence (Fig. 1D). The predicted amino acid sequence contained five C2H2-type zinc fingers that most closely resembled those of the mammalian GLIS family, which is closely related to the Gli and Sox families (Fig. S2). The pc/glis3 protein had 53% and 51% homology to human and mouse GLIS3, respectively.

We identified two *pc/glis3* mRNA variants in medaka kidney (Fig. 1D). The third exon of the *pc/glis3* gene was alternatively spliced to produce two different mRNAs with two different presumptive start codons (AB353137, AB353138), and thus, two different putative proteins (783 and 585 amino acids). Both forms were detected at all developmental stages and in all tissues examined (data not shown).

In the pc mutant, the 3′ region of the *pc/glis3* transcript was replaced by several different sequences, possibly resulting from alternative splicing of a common genomic region (Fig. 1E and Fig. S3). On the basis of the cDNA sequences, we predicted that the resulting defective *glis3* products had a normal N-terminal domain and one intact zinc finger, but that they lacked a normal C-terminal domain including the other four zinc fingers (Fig. 1G). Further analyses of the pc mutant genome revealed that intron 4 in the pc mutant contains an insertion consisting of a transposon-like sequence which has ends that are similar to those found in the medaka rs-3 mutant (Fig. 1F and Fig. S4)[37]. The medaka rs-3 mutant, in which scales are lost from the body surface, has been shown to have a transposon in the first intron of the ectodysplasin-A receptor, which causes a splicing defect. Similarly, in the pc mutant, the insertion is transcribed and spliced to produce aberrant *pc/glis3* mRNA encoding a defective glis3 protein lacking a complete zinc finger domain and C-terminus (Fig. S5).

### pc mRNA exists in the renal epithelial cells of the pronephros and mesonephros

**Figure 2:**
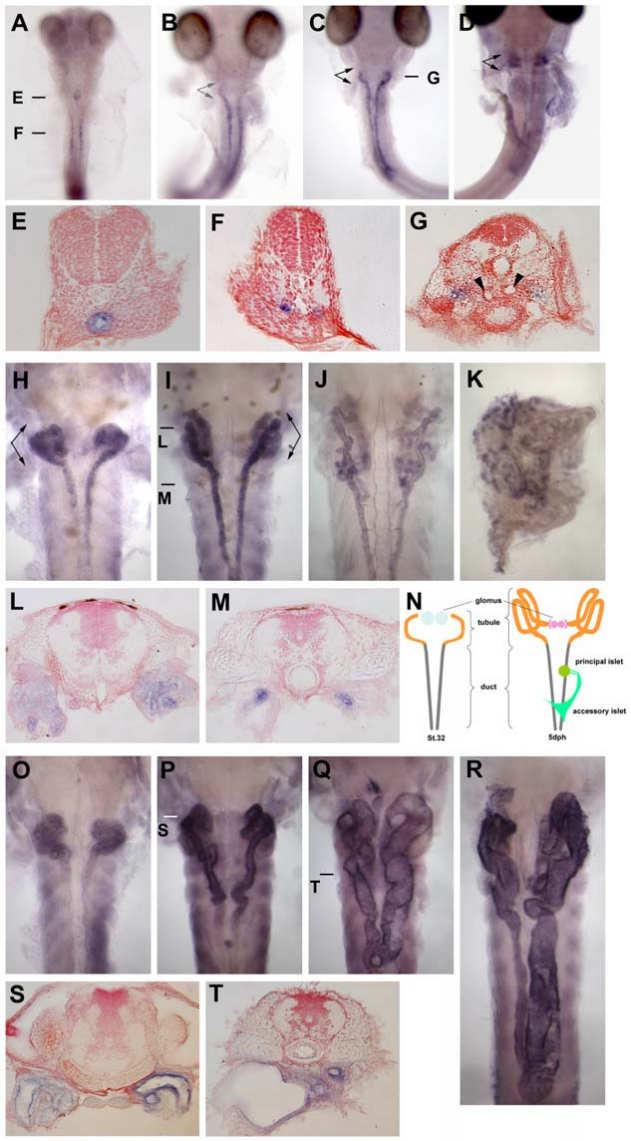
*pc/glis3* expression in medaka kidney. The expression pattern of *pc/glis3* mRNA in embryonic kidney of wild-type orange red medaka is shown for larvae of (A) stage 27, (B) stage 30, (C) stage 32, and (D) stage 35. In sections (E) and (F) (stage 27) and (G) (stage 32), *pc/glis3*-positive cells can be observed in the epithelium of the gut, the renal duct, and the renal tubule, respectively. Arrowheads in (G) indicate the pronephric glomus. *pc/glis3* expression in larval kidney of wild-type medaka is shown for (H) the hatching stage, (I) 5 days posthatching (dph), (J) 10 dph, (K) 20 dph, and (L, M) sections of the 5 dph fry in (I). In (N), segments of the kidney and the pancreas at stage 32 (left) and at 5 dph (right), are shown [33]. At stage 32, developing pronephric glomera (blue) are located most anteriorly, followed by the pronephric tubules (orange) and ducts (gray). The pronephric tubules and glomera develop after the pronephric ducts have formed [33]. By 5 dph, the pronephric glomera (pink) and tubules (orange) have become mature. The pancreatic principal (green) and accessory (dark green) islets can be visualized by in situ hybridization with *insulin*. Expression of mutant *pc/glis3* mRNA in pc mutant kidney is shown for (O) the hatching stage, (P, Q) 5 dph, (R) 10 dph, and (S, T) 5 dph. The pc mutant individual shown in (Q) and (T) has more severe dilation of the renal tubules and ducts than that in (P) and (S). Arrows in (B), (C), (D), (H), and (I) indicate the positions of tubular segments. Bars in (A), (C), (I), (P) and (Q) indicate the positions of the sections shown in (E), (F), (G), (L), (M), (S) and (T). The sections were stained with neutral red to visualize the cell nuclei.

Development of the pronephros in medaka becomes histologically apparent at the mid-somite stages (stage 25) when the pronephric duct can be first recognized. This stage is followed by development of the tubule at stage 31, and the glomus at stage 35 (Fig. 2N) [33]. By 5 dph, the tubular segment has formed multiple loops and, thereafter, mesonephric nephrons start to appear around the loops.

We examined *pc/glis3* mRNA expression during kidney development in medaka using in situ hybridization. *pc/glis3* transcripts were first detected at stage 27 (24-somite stage) in part of the developing duct of the pronephros (Fig. 2A, F) and in the anterior part of the developing foregut, which had an obvious lumen (Fig. 2A, E). At stage 30 (35-somite stage), the entire length of the developing duct was positive for *pc/glis3* mRNA (Fig. 2B), but no expression was detected in the tubular region. Sectioning revealed that the positive signal localized specifically in the renal epithelia of ducts, in the tissue facing the lumen (Fig. 2F). The tubular segment that connects the duct with the forming pronephric glomus started to express *pc/glis3* mRNA from stage 32 (somite-completion stage) (Fig. 2C, G, N). At stage 35 (when formation of the pronephros is completed) this tubular segment stained more strongly for *pc/glis3* than the duct (Fig. 2D). After hatching, *pc/glis3* mRNA expression was maintained in the renal tube structures, the tubule, and the duct (Fig. 2H, I, L, M, N). Beginning at 10 dph, mesonephric nephrons develop mostly in the tissue surrounding the pronephric tubule (the anterior portion of the pronephros) [33] and thus, accordingly, *pc/glis3*-positive mesonephric tubules were visualized mainly in the anterior field of the kidney (Fig. 2J, K).

In the pc mutant, despite the abnormal 3′ region, *pc/glis3* mRNA was expressed as in wild-type medaka (Fig. 2O-T). In situ hybridization using the 5′ region or the transposon-derived region as a probe revealed that the expression pattern of *pc/glis3* was normal in the pc mutant (Fig. 2O). Transcripts were undetectable in mutants when a wild-type 3′ probe was used (data not shown). At all stages examined, the pc mutants exhibited positive staining in the renal epithelial cells (Fig. 2O-R and data not shown). Subsequently, when dilation of the renal tube structures had been initiated (5 dph), *pc/glis3* mutant mRNA expression was observed in a thin layer of the epithelial cells lining the enlarged lumen (Fig. 2S, T).

These observations show that the *pc/glis3* expression domain is consistent with manifestation of the pc phenotype, supporting our hypothesis that *pc/glis3* is the causal gene of the pc mutation.

### Knockdown of *pc/glis3* results in pronephric cyst production

**Figure 3:**
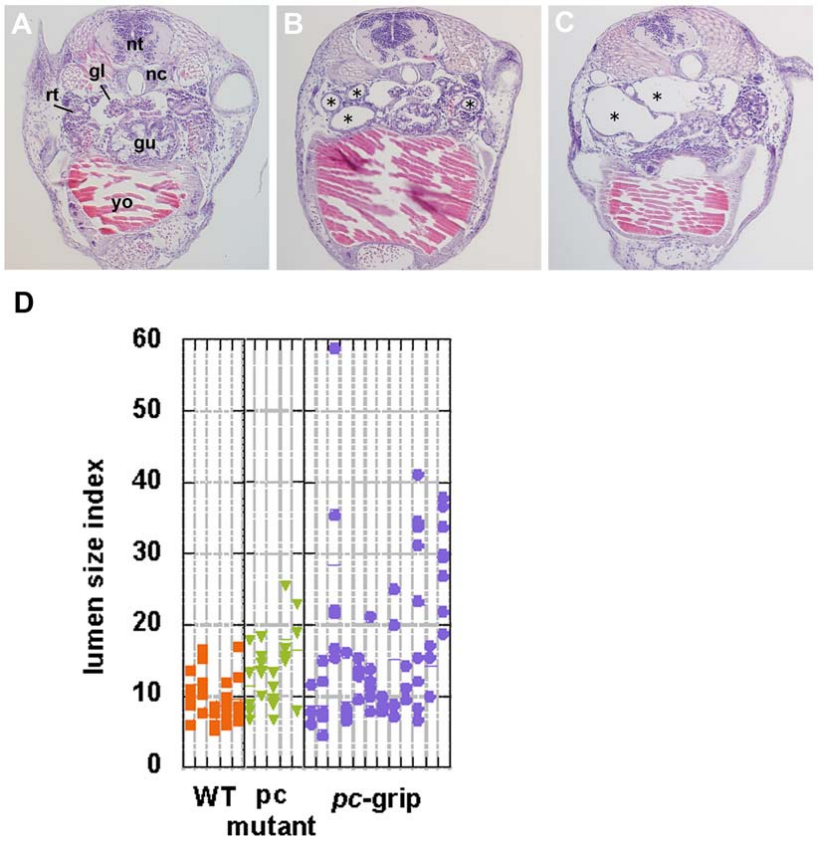
Cyst formation in *pc/glis3* knockdown individuals. Gene-specific knockdown with antisense oligonucleotides resulted in dilation of the renal tubules (B), ducts (not seen in this figure), and glomera (C). In (A), a cross section of a hatching fry with no notable dilation is shown. gl, glomus; gu, gut; nc, notochord; nt, neural tube; rt, renal tubule; yo, yolk. Typical dilation of the tubule is indicated by a star (B, C). The size of the lumen of the renal tubule was measured and is presented as a lumen size index (D). The lumen size index was defined as being a perpendicular bisector of the greatest tubule diameter of the lumen in a section. This was done in order not to overestimate the lumen size in the event that the lumen was sectioned at a right angle or the lumen shape was oval. Mean lumen size index values are indicated by a horizontal bar and measurements were taken for several sections of the tubule (arrows in Fig. 2H) at locations where the glomus was observed in the hatchlings. The lumen size indices for each individual are shown in a single lane, with individual dots in each lane representing the lumen size index value of a single tubular section. Results from knockdown with *pc*-grip (*pc*-SPD) in wild-type medaka as well as from pc mutants are shown. Compared to untreated wild-type medaka, the lumen size index in *pc/glis3*-knockdown fry is significantly higher (p<0.005).

To confirm that the *glis3* gene is involved in PKD, we explored whether antisense-directed knockdown of *glis3* led to cyst formation. A series of antisense oligonucleotides, which targeted the two alternative start codons (*pc*-ATG-1, -2) and the fourth splicing donor site (*pc*-SPD), were designed. Medaka hatchlings that had been treated with the antisense oligonucleotides had severe dilation (4/12) of the pronephric tubule and/or duct (Fig. 3A, B), and sometimes of Bowman's capsule (Fig. 3C). The diameter of the lumen in knockdown fry was 3–4 times larger than in wild-type fry (Fig. 3D). No marked differences were apparent in the knockdown effects of the three antisense oligonucleotides, and the renal phenotypes of the knockdown and pc mutant fry were relatively similar. The findings indicated that *pc/glis3* is involved in PKD and that the mutation in pc mutants, which leads to the truncation of some zinc fingers and the C-terminus, results in a loss of function in the pc/glis3 protein. Our observation that some of the knockdown fry exhibited more severe and earlier onset dilation of the renal tubules than pc mutants implies that the mutation in pc mutants may be a hypomorphic allele.

### Medaka *glis3* expression in the pancreas

**Figure 4:**
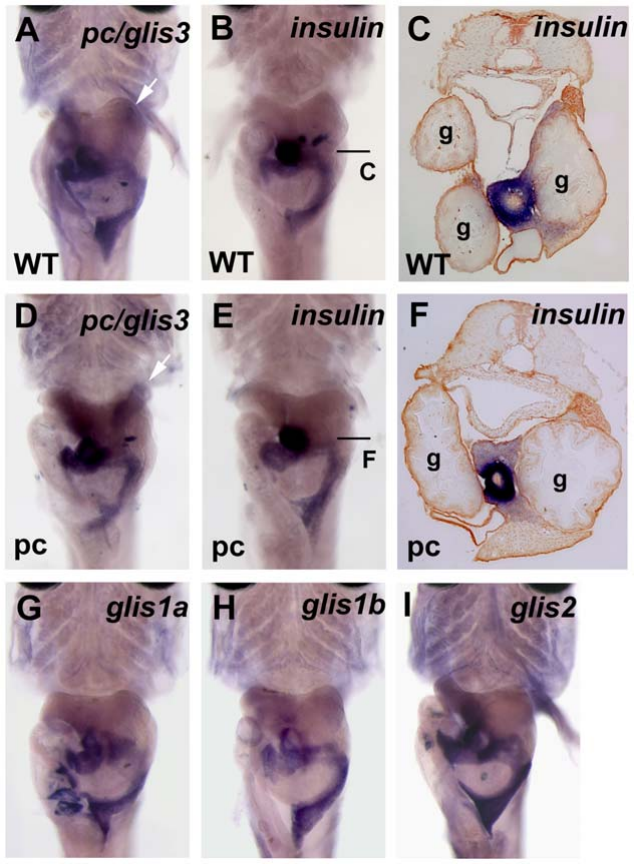
*pc/glis3* expression in the medaka pancreas. In situ hybridization was performed using 5-day posthatching fry with intact internal organs. Compared with Fig. 2N, the principal and accessory islets of the pancreas can be easily seen to be positive for the expression of all genes shown. The pancreas often varies in shape. White arrows indicate staining in the renal tubules. Arrowheads indicate the principal islets. (A, B, C) wild-type. (D, E, F) pc mutant. (A, D) *pc/glis3*, (B, C, E, F) *insulin*, (G) *glis1a*, (H) *glis1b*, and (I) *glis2* expression was detected. (A, B, D, E, G, H, I) ventral view. The principal islet sections show that there is no apparent difference in the number of *insulin*-positive cells (C, F).

For comparison between species, we examined expression of the *pc/glis3* gene using in situ hybridization. In medaka fry, the *pc/glis3* gene was expressed in the pancreas. The distribution of *pc/glis3*-positive cells was similar to the distribution of *insulin*-positive cells (Fig. 4A, B, C, G, H, I), implying that, like the human *GLIS3* gene, the medaka *glis3* gene might cause pancreatic defects. However, pancreatic *insulin* expression was not affected in the pc mutant (Fig. 4D, E, F).

### Other *glis* family members in medaka

In humans and mice three *GLIS* family genes, *GLIS1*, *GLIS2*, and *GLIS3*, have been found [24], [38]–[41]. A search for genes belonging to the *GLIS* family in the medaka genome revealed four genes, the cDNA sequences of which were used to construct a phylogenetic tree with clustalW (Fig. S2). The tree revealed that medaka have two copies of *glis1*, one copy of *glis2*, and one copy of *glis3 (pc)*. We then examined the expression of each *glis* gene using in situ hybridization and found that all of the *glis* genes including *pc/glis3* were expressed in the pancreas of medaka fry, and that the cells in which *glis* expression occurred overlapped with the distribution of *insulin*-positive cells. In contrast, none of the *glis* genes other than *pc/glis3*, was detected in the kidney (pronephros) of medaka fry (Fig. 4A, D).

### Impaired urine flow rate and shortened renal cilia in the pc mutant

**Figure 5:**
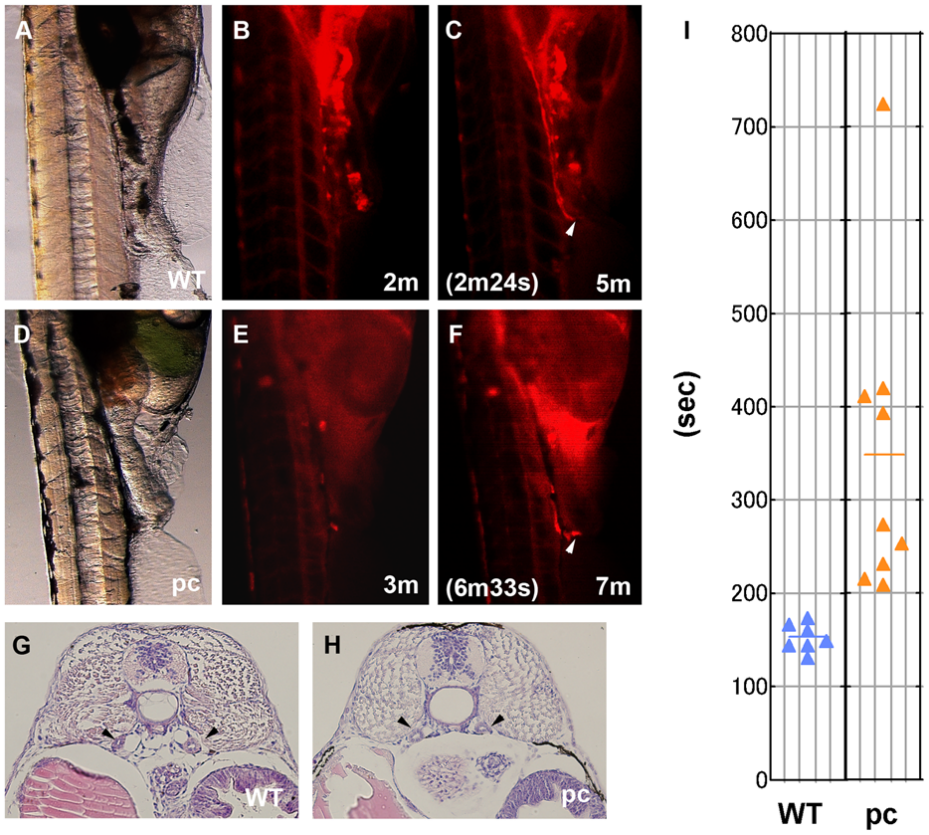
Urine flow in the pc mutants. At 10-day postfertilization (dpf), 5% rhodamine-conjugated dextran was injected into the common cardinal vein of medaka fry. The dye was filtered in the pronephric glomera, passed through the pronephric tubules and ducts, and subsequently excreted into the bladder. (A, B, C) Wild-type fry, (D, E, F) pc mutant fry. (A, D) are transmitted light images, (B, E) are fluorescence images obtained before the dye was excreted into the bladder, at 2 min after dye injection in the wild-type and at 3 min after dye injection in the pc mutant. (C, F) Fluorescence images obtained after dye excretion, 5 min after dye injection in the wild-type and 7 min after dye injection in the pc mutant. Arrowheads indicate the positions of dye excretion into the bladder. In the samples shown, dye excretion was first observed at 2 min 24 s in the wild-type and at 6 min 33 s in the pc mutant. (G, H) Cross sections of the trunk region of the fry showing that ductal distension has not yet occurred in the pc mutant and thus cannot be affecting the urine flow rate. Arrowheads indicate the positions of pronephric ducts. (I) The time after injection until first entry of fluorescence into the bladder was 153.0±14.7 s in wild-type fry (n = 7) and 348.0±164.9 s in pc mutants (n = 9). Time to excretion for each individual is indicated by a triangle (seconds). Horizontal bars show mean values.

In numerous PKD models, pathological features are attributed to the absence of cilia or kidney dysfunction [2]. Since the ciliary defects in some zebrafish and medaka PKD models are manifested as impaired urine flow rate [13], we performed dye excretion experiments to determine whether pronephric urine flow was normal in pc mutants. Rhodamine-conjugated dextran was injected into the common cardinal vein of living 1-day old fry (10 dpf), and was observed to be filtered in the pronephric glomus and excreted via the pronephric ducts into the urinary bladder. The time after injection before the urine first became visible in the bladder was 153.0±14.7 s in wild-type fry (Fig. 5). In the pc mutant, the corresponding time was significantly longer (348.0±164.9 s). The delay in the dye reaching the bladder was not due to decreased blood circulation, because the heart rate of the pc mutants was comparable to that of wild-type individuals (approximately 90 beats per minute). In addition, the lumens of the pronephric tubules and ducts in pc mutants and wild-type individuals were the same size (Fig. 5G, H), suggesting that the reduction in urine flow rate was not due to dilation of the tubules or ducts.

**Figure 6:**
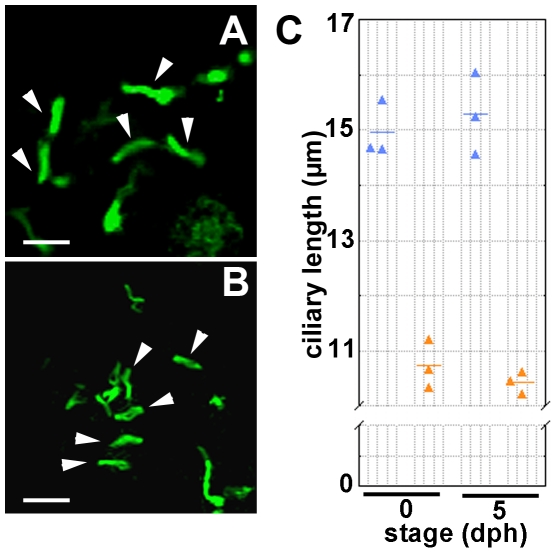
Length of renal cilia. The renal cilia of medaka hatchlings were visualized by immunofluorescence analysis using anti-acetylated alpha-tubulin antibody. Confocal fluorescence images of the cilia in tubular segments are shown for (A) wild-type individuals and (B) pc mutants. Measurement results of cilia length are shown in (C); the cilia were significantly shorter in pc mutants (10.73±0.44 µm, n = 3, at 0dph and 10.43±0.21 µm, n = 3, at 5dph, blue triangles) than in the wild-type (14.97±0.51 µm, n = 3, at 0dph and 15.28±0.74 µm, n = 3, at 5dph, orange triangles)(p<0.005). Data show the mean lengths for at least 15 cilia in each individual. Horizontal bars show the means of the specimens. Arrowheads indicate examples of cilia measured. Triangles show the mean cilia length (µm) for each individual. Scale bars show 20 µm.

To investigate why the urine flow decreased in pc mutants, we examined the motility of renal cilia. In kidneys from the pc mutants, the cilia beat in a circular motion with a constant frequency (data not shown) that was indistinguishable from that observed in the wild-type. We also examined the length of the renal cilia by visualization with anti-alpha-tubulin (Fig. 6A, B). We previously reported that the pc mutant had renal cilia in the cystic mesonephros [15]. Although we did not determine the ciliary length in the previous study, the SEM images of the mesonephric cilia were apparently normal in the pc mutant adult fish. Length measurement of the cilia in zebrafish has shown that the cilia in different organs vary in length [13]. We also noticed that the ciliary length differs dependent on segmental positions: the cilia in the pronephric duct are much shorter than those in the tubule (data not shown). In this study, the lengths of the cilia in the tubular portion of the pronephros were compared between the wild-type and the pc mutant fries. Our measurements revealed that the cilia were significantly shorter in pc mutants (10.43±0.21 µm) than in wild-type fish (15.28±0.74 µm) (Fig. 6C). These results implied that the abnormally short cilia in pc mutants were responsible for the relatively decreased rates of urine flow compared to wild-type kidneys, and that this led to the accumulation of urine and distension of the tubules and ducts in the pronephros.

### Proliferation of renal epithelial cells may not be the direct cause of cyst formation

**Figure 7:**
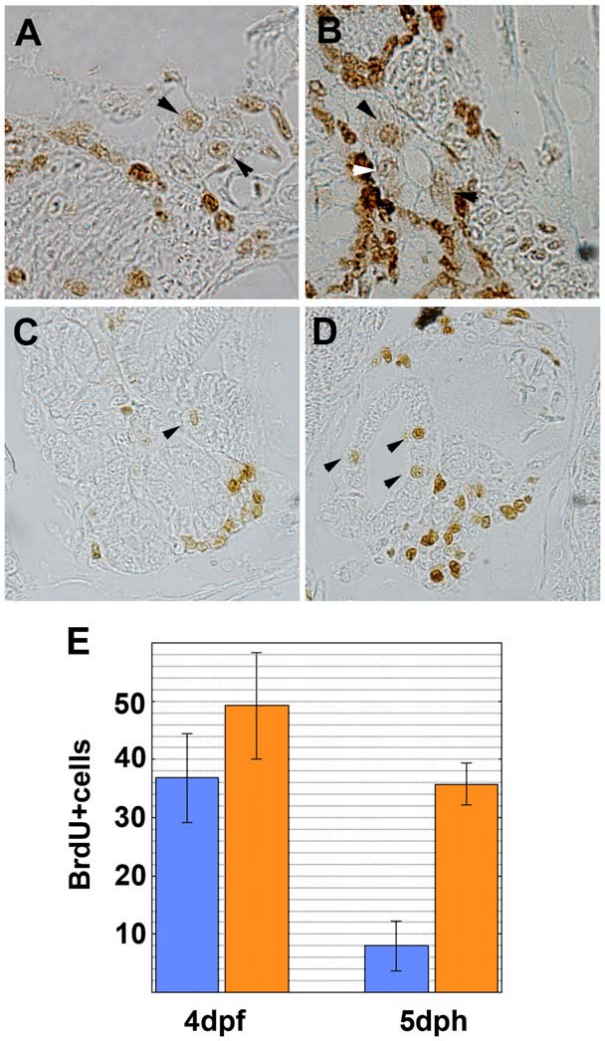
Cell proliferation in the renal tubules and ducts. Cell proliferation in the kidney was compared between (A, C) wild-type and (B, D) pc mutant medaka at 4-day postfertilization (dpf) (A, B) and 5-day posthatching (dph)(C, D). Arrowheads indicate BrdU-positive cells in the renal ductal or tubular epithelia. (E) Number of BrdU-positive cells in the epithelium of the anterior portion (most anterior 15 sections) of the pronephros. Sections were obtained from four wild-type and four pc mutant fry at both 4 dpf and at 5 dph. The number of BrdU-positive cells was significantly different in wild-type (blue) and pc mutant (orange) medaka at 5 dph (p<0.005), but not at 4 dpf.

Cystogenesis in PKD is believed to be closely related to the proliferation of the renal tubular epithelial cells [2], [42]. To investigate the possibility that *pc/glis3* is involved in renal tubular cell proliferation, we performed BrdU labeling of medaka embryos and fry. The number of BrdU-positive cells in the tubular or ductal epithelium of the developing pronephros of 4 dpf embryos, as well as in the functional pronephros of 5 dph fry, was counted for a number of sections (Fig. 7). At 4 dpf, before development of the pronephros is complete, both wild-type and pc mutant embryos had a number of BrdU-positive cells in the tubular and ductal segments of the developing pronephros. No significant difference in the number of BrdU-positive cells was observed between the wild-type and the pc mutant (Fig. 7A, B). However, we detected significant cell proliferation in the pronephric tubules of pc mutant fry at 5 dph (Fig. 7D, 35.8±3.6 positive cells in the anterior portion of the pronephros, n = 4), while there were few proliferating tubular epithelial cells in the pronephros of wild-type fry (Fig. 7C, 8.0±4.2 positive cells, n = 4). Given that *pc/glis3* expression is specific to the renal tubular cells, it is possible that the *pc/glis3* mutation caused elevated cell proliferation in the renal tubules. However, increased cell proliferation did not precede the occurrence of urine flow reduction in the pc mutant, implying that elevated cell proliferation was not the primary phenotype causing cyst formation.

### pc/glis3 protein is localized in primary cilia

**Figure 8:**
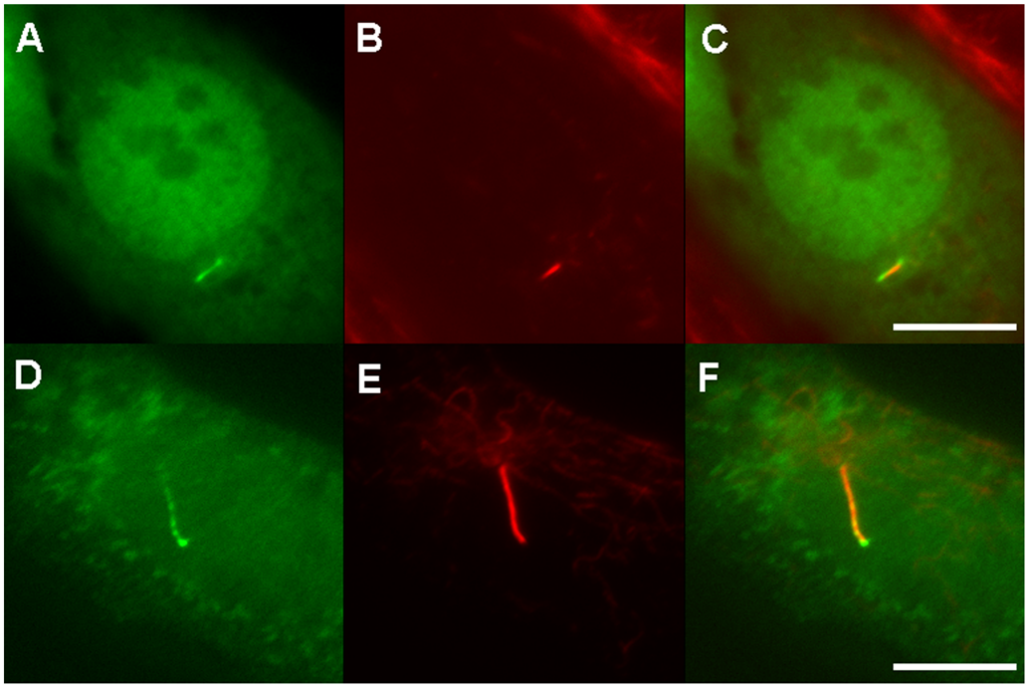
Subcellular localization of the pc/glis3 protein. EGFP-tagged pc/glis3 protein fluorescence was detected along the entire length of the cilium in mouse cultured renal epithelial cells (A-F). (A, D) EGFP-fluorescence. (B, E) acetylated alpha-tubulin, used as a cilium marker (Alexa555). (C, F) Merged pictures. The pc/glis3 fluorescent signal was also detected in the nucleus (A, C). Scale bars show 10 µm.

To further investigate if *pc/glis3* is involved in the function of renal cilia, we examined the localization of the pc/glis3 protein in renal epithelial cells. For this purpose a GFP-tagged pc/glis3 construct was introduced into the mouse renal epithelial cell line (Dai1 cells) [36]. pc/glis3-GFP fluorescence was observed in a punctuate fashion throughout the entire ciliated region (Fig. 8). The immunohistochemical signal from acetylated alpha-tubulin almost completely overlapped with the pc/glis3-GFP fluorescent signal, suggesting that the pc/glis3 protein was localized in the cilia. In a large number of cells, pc/glis3-GFP fluorescence was also detected in the nucleus, indicating that the nuclear localization signal in the carboxy-terminal domain of pc/glis3 was functional and that the pc/glis3 protein preferentially sorts to both the nucleus and the cilia under our experimental conditions.

## Discussion

### A putative role for *pc/glis3* in PKD pathogenesis

In this study, we have shown that a mutation in *pc/glis3* causes PKD in medaka (Fig. 1). *pc/glis3* is continuously expressed in the ciliated epithelial cells of the renal tubule and duct during pronephric and mesonephric development (Fig. 2), and knockdown of *pc/glis3* leads to dilation of the pronephric tubules (Fig. 3). Taken together, these findings indicate that *pc/glis3* is involved in the maintenance and/or regulation of renal tubules.

Numerous previous studies on human and rodent cystic kidney disease have indicated that PKD is a cilia-associated disease [1], [2], [10], [43]–[51]. In mammals, the cilia on the surface of renal epithelial cells are believed to be immotile, acting merely as mechanosensors of urine flow to maintain the lumen of the renal tubule at an appropriate size. Disturbed ciliary structure or function results in PKD [6], [50], [52], [53]. We previously reported the occurrence of cilia on the surface of the renal tubular and ductal epithelium in medaka [15]. However, unlike the 9+0 arrangement of mammalian renal cilia, the cilia in medaka are organized in a 9+2 arrangement, the same arrangement as that usually found in motile cilia [2], [13], [15]. In fact, in the present study we found that medaka renal cilia are motile, beating in a circular motion and having an undulating appearance (data not shown). Previous studies on zebrafish PKD mutants have shown that disruption of the structure or motility of cilia results in pronephric cyst formation [2], [13]. In the pc mutant, renal cilia have been observed in the renal tubules and ducts [15]. Our present data show that cilia were significantly shorter and urine flow rate was reduced in the pc mutant. We speculate that the abnormally short cilia of renal epithelial cells may not be able to generate the driving force required for normal rates of urine flow, possibly leading to urine accumulation and subsequent cyst formation. Urine accumulation or cyst formation may cause the secondary phenotype of elevated cell proliferation in the tubular and ductal cells (Fig. 7). Despite the existence of inter-species differences in the architecture and motility of renal cilia, our results indicate that having cilia of sufficient length is crucial for maintaining lumen size in the renal tubules and ducts. Loss of *pc/GLIS3* function in medaka and humans results in renal cyst formation, implying that GLIS3 is functionally equivalent in these animals.

Further evidence implying a link between the cilia and PKD pathogenesis in medaka pc mutants is derived from the analysis of the subcellular localization of the pc/glis3 protein. Our data show that the EGFP-tagged pc/glis3 protein was localized in the cilia and the nuclei of mouse renal epithelial cells (Fig. 8). Similarly, Attanasio et al. have recently shown that the GLIS2 protein is localized in both the cilia and the nuclei in cultured canine kidney (MDCK) cells [41]. Members of the GLIS family structurally resemble Gli transcription factors, which act downstream of Hedgehog [54]. It has recently been reported that Hedgehog signaling requires cilia for activation of downstream genes [55]–[62] and that the Gli proteins Gli2 and Gli3 are localized at the tips of cilia before translocation to the nuclei in response to Hedgehog ligand-receptor binding [62]–[64]. The similarity between GLIS and Gli proteins tempts us to speculate that *pc/glis3* is involved in a signal transduction pathway, in a manner similar to Ci/Gli in Hedgehog signaling.

### Are pc mutants really analogous to patients with a *GLIS3* mutation?

In humans and mice, three *GLIS* genes (*GLIS1*, *GLIS2* and *GLIS3*) have been found [38]–[40], [65]. Comparison of the primary structures of these genes suggests that medaka have two *glis1* counterparts (Fig. S2). By positional cloning, we found that *RFX3* is located in the flanking region of *pc/glis3*. Interestingly, as a result of searching the genome databases of other species, we found that *RFX3* or other *RFX* isoforms, is not adjacent to other members of *glis* family in all species examined (humans, mice, zebrafish and pufferfish). This evolutionary conservation of *pc/glis3* and *RFX3* synteny indicates that *pc/glis3* is equivalent to human and mouse *GLIS3*.

Senee et al. determined that mutations in *GLIS3* are responsible for a rare syndrome with a pleiotropic phenotype that includes neonatal diabetes mellitus, congenital hypothyroidism, and cystic kidney [24]. Three distinct alleles of *GLIS3* are involved in this syndrome [24], [66]. One allele, *NDH1*, which harbors a single base pair insertion that causes a frameshift mutation and a truncated protein, cause cystic kidney, while the other two alleles, *NDH2* and *NDH3*, which have large deletions in the *GLIS3*-flanking region, do not. The mutation found in *pc/glis3* of the medaka pc mutant was caused by a large transposon inserted at a position corresponding to the second zinc finger, which we predict would result in a defective zinc finger motif and the absence of the C-terminal region (Fig. 1G). Truncation of the C-terminal region of GLIS3 has also been shown to inhibit its transcriptional promoting activity in vitro [40]. These lines of evidence, together with our results, suggest that the C-terminal domain of pc/glis3 is crucial for the proper function of this protein in the kidney. Despite the allele-specific manifestation of cyst formation in human patients, our finding that *pc/glis3* causes PKD in medaka has clearly shown that loss of GLIS3 function can lead to renal cyst formation across species.

Transcripts of *pc/glis3* were observed to be abundantly expressed in the beta cells of medaka in this study. However, no mutant phenotypes were found in the pancreatic tissues of pc mutants. Conversely, human *GLIS3* mutations cause neonatal diabetes. Further studies are required to explain the functional differences in the pancreas between medaka and humans. Notably, there are fundamental differences in the nutritional requirements of humans and fish; for example, amino acids rather than glucose are the most important insulinotropins in fish [67]. Consequently, the pathogenesis of diabetes may differ markedly in mammals and fish. However, if it is assumed that members of the *glis* family have similar activities, the overlapping expression of the different *glis* family members in the pancreas but not in the kidney might preclude the occurrence of pancreatic phenotypes in the pc mutant. Ultimately, this difference in phenotype between mammals and medaka would position the pc mutant as a kidney-specific disease model of human *GLIS3* mutations.

In conclusion, we identified the medaka pc gene, an ortholog of human *GLIS3*, as a gene causing cystic kidney. The protein encoded by the gene is possibly required for normal motility of cilia located on the renal tubular and ductal epithelium and thus also for the generation of normal urine flow in medaka. To better understand the function of *pc/glis3* in the kidney and its role in PKD pathogenesis, comparative studies of the involvement of pc/glis3 in ciliary motility in fish and ciliary mechanosensitivity in mammals should be undertaken.

## Supporting Information

Figure S1 Analysis of pc/glis3 mRNA expression (A) RT-PCR of wild-type (WT, left) and the pc mutant (right) pc/glis3 mRNA. pc/glis3 mRNA was not detectable in the pc mutant using the PCR primers 5′-GTTTGAAGGCTGCAAGAAGGCATT-3′ (sense) and 5′-CTTGCGTAACTGTCGCTCTTG-3′ (antisense), which would amplify the 3′ region of the transcript (271 bp). RFX3 is a neighboring gene of pc/glis3 and EF-1alpha acted as a control. For RFX3 mRNA detection, the PCR primers 5′-CGTTGCGCAGATATACGTCAC-3′ (sense) and 5′-AGGCGTCTCTCCAGTAGCTTG-3′ (antisense) were used. EF-1a mRNA detection was conducted as previously described [68]. (B) Northern blot analysis pc/glis3 mRNA was detected in WT kidney but not in pc kidney or WT liver by northern blot analysis using the fragment of glis3 cDNA containing exons 3–6 as a probe. The mRNA was approximately 4 kb in length. The smeared signals in the pc kidney sample may be due to aberrantly spliced RNAs of various lengths. Indeed, we identified numerous RNAs in the pc mutant kidney that have different exons derived from inserted genomic regions. These RNAs contained a variety of 3′ pc mutant-specific exons in their 3′ tail regions.
(0.19 MB TIF)

Figure S2 The sequence of pc/glis3 cDNA and its deduced protein. The pc/glis3 cDNA sequence is shown in the upper line and its deduced amino acid sequence in the lower line. The exon boundaries are indicated by color: the 3′ end of the exon in front is in red and the 5′ end of the exon behind is in blue. The position of the probe used for northern hybridization is indicated by underlining. The region amplified by RT-PCR (Fig. S1A) is indicated by double-underlining. The region of alternative splicing (73 bp) in exon 3 is shadowed in gray. The two alternative start codons are shadowed in green.
(0.04 MB DOC)

Figure S3 The phylogenetic tree constructed for Kruppel-like transcription factors Shown are pc/glis3 (AB353137), glis1a (AB353139), glis1b (AB353140), and glis2 (AB353141) from medaka; Glis1 (NP_671754), Glis2 (Q8VDL9), Glis3 (ABI31654), Gli1 (P47806), Gli2 (NP_001074594), Gli3 (Q61602), Zic1 (AAH6024), Zic2 (Q62520), and Zic3 (Q62521) from the mouse; and Cubitus interruptus (Ci, P19538) from Drosophila. The tree was drawn using the ClustalW program, and was constructed based on the complete amino acid sequence.
(0.19 MB TIF)

Figure S4 Structure and expression of pc/glis3 mRNA (A) Structure of the pc/glis3 mRNA The structure of the WT and pc pc/glis3 mRNA is described in detail in the legend to Fig. 1G. (B) RT-PCR of the 3′ region of pc/glis3 mRNA An exon 4 fragment was amplified from both the WT and pc mutant medaka by RT-PCR with the primer set described in the text and detailed below (a). The 3′ region extending over exon 4 and exon 5 was not obtained in the pc mutant (b), although the pc mutant mRNA had a distinct exon 5 that was not detected in WT (c). Primers used: (a)5′-CACTGCTGCCGATGGATGGACTG-3′ /5′-TTGTTGGGCTTCTCTCCAGAATG-3′ (b)5′-CACTGCTGCCGATGGATGGACTG-3′ /5′-CTTGCGTAACTGTCGCTCTCTT-3′ (c)5′-CACTGCTGCCGATGGATGGACTG-3′ /5′-GCCAGAGCTGTCTGCTGTGACG-3′
(0.17 MB TIF)

Figure S5 RT-PCR of the 3′ region of pc/glis3 mRNA (A) The regions targeted for PCR amplification (B) RT-PCR of WT and mutant mRNA (C) Genomic PCR of the regions corresponding to those amplified by RT-PCR Primers used: (1) 5′-TTTACCACTGGGAAGCAAAGC-3′/5′-GCATGAAACTCTGCGGTATATC-3′ (2)5′-TGAGTGTTGCAACAGGCCATC-3′ /5′-CAGCATCTTCCTGGACTGTGG-3′ (3)5′-TTTGAAGGCTGCAAGAAGGCATT-3′ /5′-AACAATCTGTAAGTGTGTCCAG-3′ (4)5′-TTTGAAGGCTGCAAGAAGGCATT-3′ /5′-ATTTGTGCCATGGTCCAAAACAG-3′
(0.25 MB TIF)
