# Supplementary figures and images for: Polycystic Kidney Disease in the Medaka (Oryzias latipes) pc Mutant Caused by a Mutation in the Gli-Similar3 (glis3) Gene
Source: PLoS One. 2009 Jul 17;4(7):e6299. doi: 10.1371/journal.pone.0006299 (PMC2706989; doi:10.1371/journal.pone.0006299)

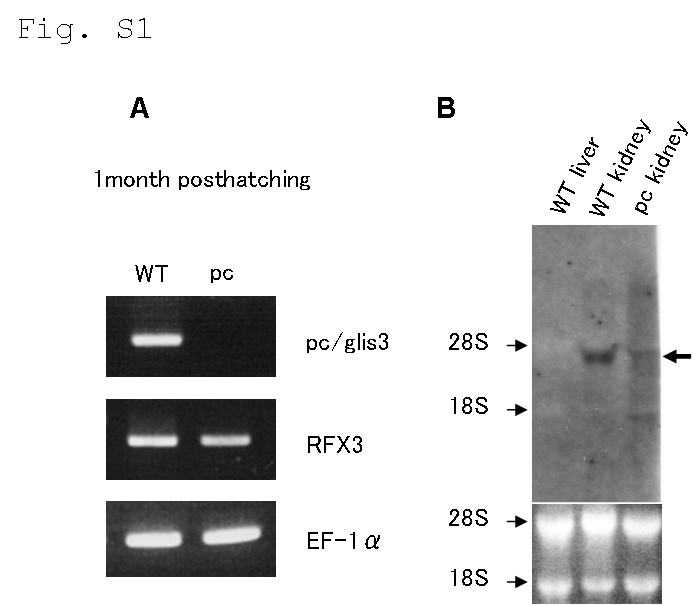

Supplement: Figure S1 — Analysis of pc/glis3 mRNA expression (A) RT-PCR of wild-type (WT, left) and the pc mutant (right) pc/glis3 mRNA. pc/glis3 mRNA was not detectable in the pc mutant using the PCR primers 5′-GTTTGAAGGCTGCAAGAAGGCATT-3′ (sense) and 5′-CTTGCGTAACTGTCGCTCTTG-3′ (antisense), which would amplify the 3′ region of the transcript (271 bp). RFX3 is a neighboring gene of pc/glis3 and EF-1alpha acted as a control. For RFX3 mRNA detection, the PCR primers 5′-CGTTGCGCAGATATACGTCAC-3′ (sense) and 5′-AGGCGTCTCTCCAGTAGCTTG-3′ (antisense) were used. EF-1a mRNA detection was conducted as previously described [68]. (B) Northern blot analysis pc/glis3 mRNA was detected in WT kidney but not in pc kidney or WT liver by northern blot analysis using the fragment of glis3 cDNA containing exons 3–6 as a probe. The mRNA was approximately 4 kb in length. The smeared signals in the pc kidney sample may be due to aberrantly spliced RNAs of various lengths. Indeed, we identified numerous RNAs in the pc mutant kidney that have different exons derived from inserted genomic regions. These RNAs contained a variety of 3′ pc mutant-specific exons in their 3′ tail regions. (0.19 MB TIF) [file pone.0006299.s001.tif]

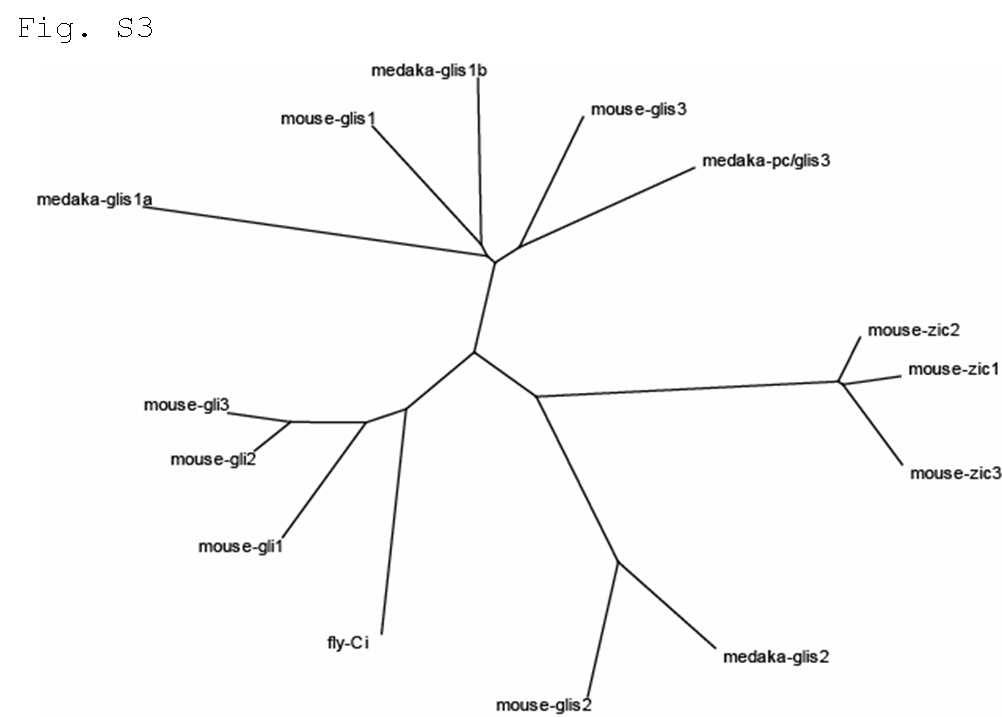

Supplement: Figure S3 — The phylogenetic tree constructed for Kruppel-like transcription factors Shown are pc/glis3 (AB353137), glis1a (AB353139), glis1b (AB353140), and glis2 (AB353141) from medaka; Glis1 (NP_671754), Glis2 (Q8VDL9), Glis3 (ABI31654), Gli1 (P47806), Gli2 (NP_001074594), Gli3 (Q61602), Zic1 (AAH6024), Zic2 (Q62520), and Zic3 (Q62521) from the mouse; and Cubitus interruptus (Ci, P19538) from Drosophila. The tree was drawn using the ClustalW program, and was constructed based on the complete amino acid sequence. (0.19 MB TIF) [file pone.0006299.s003.tif]

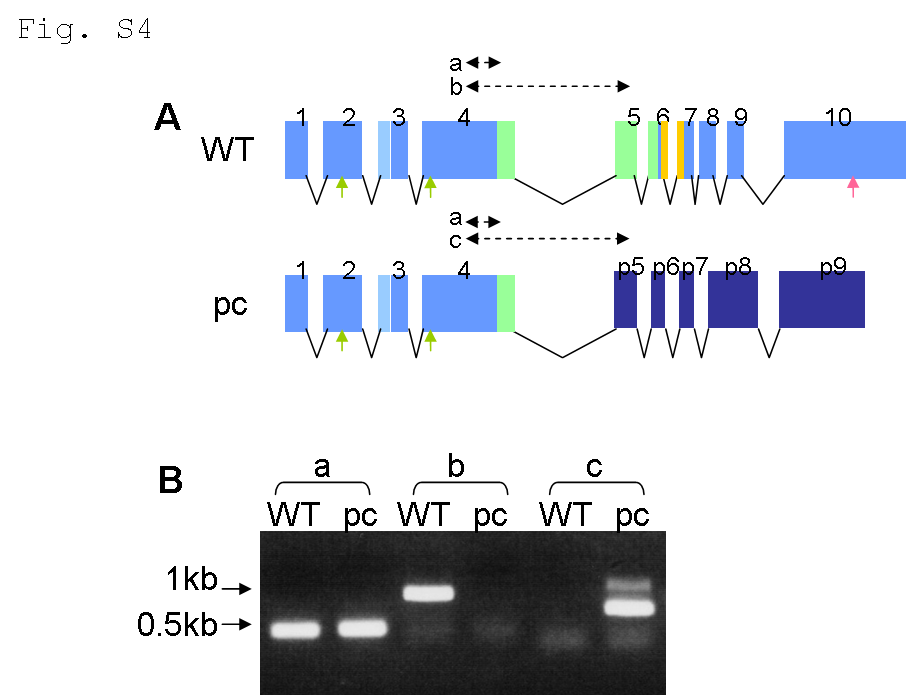

Supplement: Figure S4 — Structure and expression of pc/glis3 mRNA (A) Structure of the pc/glis3 mRNA The structure of the WT and pc pc/glis3 mRNA is described in detail in the legend to Fig. 1G. (B) RT-PCR of the 3′ region of pc/glis3 mRNA An exon 4 fragment was amplified from both the WT and pc mutant medaka by RT-PCR with the primer set described in the text and detailed below (a). The 3′ region extending over exon 4 and exon 5 was not obtained in the pc mutant (b), although the pc mutant mRNA had a distinct exon 5 that was not detected in WT (c). Primers used: (a)5′-CACTGCTGCCGATGGATGGACTG-3′ /5′-TTGTTGGGCTTCTCTCCAGAATG-3′ (b)5′-CACTGCTGCCGATGGATGGACTG-3′ /5′-CTTGCGTAACTGTCGCTCTCTT-3′ (c)5′-CACTGCTGCCGATGGATGGACTG-3′ /5′-GCCAGAGCTGTCTGCTGTGACG-3′ (0.17 MB TIF) [file pone.0006299.s004.tif]

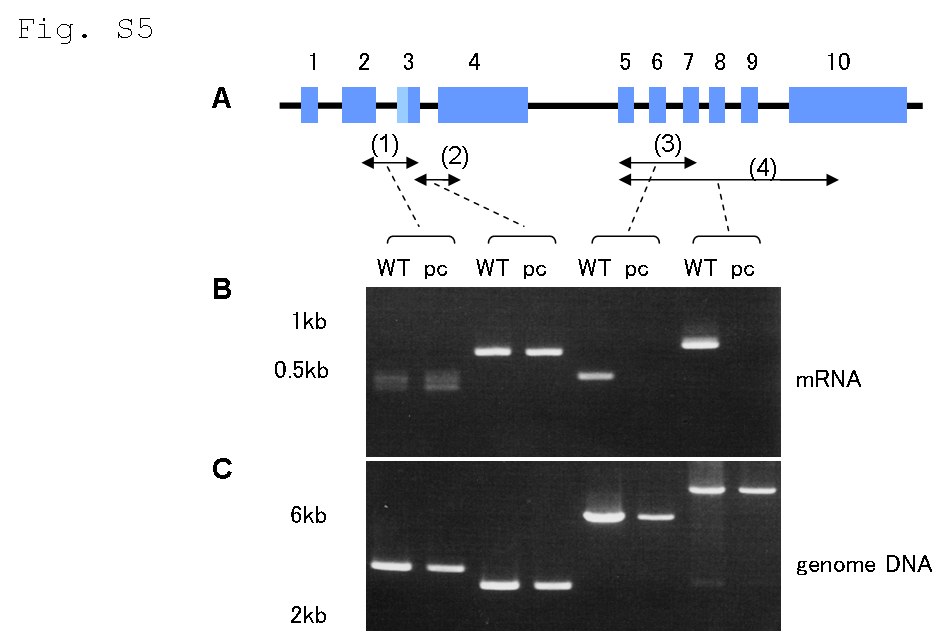

Supplement: Figure S5 — RT-PCR of the 3′ region of pc/glis3 mRNA (A) The regions targeted for PCR amplification (B) RT-PCR of WT and mutant mRNA (C) Genomic PCR of the regions corresponding to those amplified by RT-PCR Primers used: (1) 5′-TTTACCACTGGGAAGCAAAGC-3′/5′-GCATGAAACTCTGCGGTATATC-3′ (2)5′-TGAGTGTTGCAACAGGCCATC-3′ /5′-CAGCATCTTCCTGGACTGTGG-3′ (3)5′-TTTGAAGGCTGCAAGAAGGCATT-3′ /5′-AACAATCTGTAAGTGTGTCCAG-3′ (4)5′-TTTGAAGGCTGCAAGAAGGCATT-3′ /5′-ATTTGTGCCATGGTCCAAAACAG-3′ (0.25 MB TIF) [file pone.0006299.s005.tif]
