# Supplementary material for: Polycystic Kidney Disease in the Medaka (Oryzias latipes) pc Mutant Caused by a Mutation in the Gli-Similar3 (glis3) Gene
Source: PLoS One. 2009 Jul 17;4(7):e6299. doi: 10.1371/journal.pone.0006299 (PMC2706989; doi:10.1371/journal.pone.0006299)
Supplement: Figure S2 — The sequence of pc/glis3 cDNA and its deduced protein. The pc/glis3 cDNA sequence is shown in the upper line and its deduced amino acid sequence in the lower line. The exon boundaries are indicated by color: the 3′ end of the exon in front is in red and the 5′ end of the exon behind is in blue. The position of the probe used for northern hybridization is indicated by underlining. The region amplified by RT-PCR (Fig. S1A) is indicated by double-underlining. The region of alternative splicing (73 bp) in exon 3 is shadowed in gray. The two alternative start codons are shadowed in green. (0.04 MB DOC) [file pone.0006299.s002.doc]

-136 GCTGTTGAAACCTGAC -121

-120 ACAAAGATCGGACATTTGGGACAGATTGGCAAAATGGTCAATGACTTGCAGGTGTAATCC -61　　　　exon1-2

-60 CAACAATGCAAAACCTTCGATAACCCAACGACAAAACGACTTCCTGAATCCGTTTTAGAC -1

1 ATGAGTGGGAAAGGTTGTCAGCTCATGGTGTCTCCTTCCAGAGTGTCCCCACCACTAAAG 60

1 M S G K G C Q L M V S P S R V S P P L K 20

61 ATGACCAGAGGCCACCAGTCCCAGTATATCAGGATGCCGACAGCGTCCGATGACCTGGTC 120

21 M T R G H Q S Q Y I R M P T A S D D L V 40

121 AGTCTCTGCTCCTCCCTGGGGAGGTCAGAAGAAGTGAAAACGGGAAACGTGAACTCTTTT 180

41 S L C S S L G R S E E V K T G N V N S F 60

181 ACCACTGGGAAGCAAAGCGGGAGCCATGTTGTCTTGCCTGCCCTGAGTCTTCGCAGGCAA 240

61 T T G K Q S G S H V V L P A L S L R R Q 80

241 GTGCTAATCAATGGGAAACATCTAAGTGGGCCCACTGCATCCTTAGCTTCCCGTCAGCCG 300

81 V L I N G K H L S G P T A S L A S R Q P 100

301 AAACTAACAAGACTCCTCCCACCTGGCAACAGATCCAGAGATTGTGAAGTGTCTGGCTCC 360　　　　exon2-3a

101 K L T R L L P P G N R S R D C E V S G S 120

361 ACTGTGGATGTATTTGGAAAGGCAACTGATGTTAACCTCACTGTGACCAACAGTCCCCTG 420　　　　exon2-3b

121 T V D V F G K A T D V N L T V T N S P L 140

421 AGTGTTGCAACAGGCCATCGATATACCGCAGAGTTTCATGCCCAGTCGTTTCATAATGCG 480

141 S V A T G H R Y T A E F H A Q S F H N A 160

481 CCGGCTCCCCAAAGTGATGGCAGATCCCTTCTGTCCAGAGAATCCCTGGCATCCACCACC 540　　　　exon3-4

161 P A P Q S D G R S L L S R E S L A S T T 180

541 CTTAGTCTGTTTGAAACACAGTCCATGTTTAGTGGCAAACACGACTGGCCATATGGTTAC 600

181 L S L F E T Q S M F S G K H D W P Y G Y 200

601 CGTGTGCTTCCTCCCCTGGGATCATCCCACTGCTCTAACCAAGCCAACGAGGGCTGTGAG 660

201 R V L P P L G S S H C S N Q A N E G C E 220

661 CAGTTCAGTCTCTCCCCAGGCGCTGCCATGTCAGGCACAGCAAGCACCTCCGCCTCCCTC 720

221 Q F S L S P G A A M S G T A S T S A S L 240

721 CCATCATACCTTTTTGCATATGAGACTGGAAGCCCCAGACAGACAGGTGCGAGGAAGAGA 780

241 P S Y L F A Y E T G S P R Q T G A R K R 260

781 CCTCTCTCCATGTCACCTTTGTCGGATCTTATGGGTATTGACTTCAACTCTATTATACGT 840

261 P L S M S P L S D L M G I D F N S I I R 280

841 ACGTCACCCACTTCCCTTGTGGCTTATATTAATGGTCCCCGCAGTTCACCAGCCTCCTAC 900

281 T S P T S L V A Y I N G P R S S P A S Y 300

901 TCCACCGTCTCACCCATTCAGTCCGATGGATACGGTCACTTCCTGGGAGTGAGAGGTCGC 960

301 S T V S P I Q S D G Y G H F L G V R G R 320

961 TGCATACCTCAGAACCACCCCTACAGTTTTCCCGGTTCCTCCCAAATTCCAGCCACACAG 1020

321 C I P Q N H P Y S F P G S S Q I P A T Q 340

1021 TTCGAATGTGGCCGGATGGAGATGATCGAAGAGGGAAGTAGTCTGGAAAGCCAGATGGCC 1080

341 F E C G R M E M I E E G S S L E S Q M A 360

1081 AACATGGTGGTGAAGCAACAGTGCTTCCCTGAGGAAATTGGGTTTCTGGAAAAGACTACA 1140

361 N M V V K Q Q C F P E E I G F L E K T T 380

1141 GACAGCGGCAGCCAACCTAGCAACAATGTTCTTCTGTCTCTACAGCCAGAGCCAGCTGCA 1200

381 D S G S Q P S N N V L L S L Q P E P A A 400

1201 TTATCCACAGTCCAGGAAGATGCTGCTTCACTTGGGCCTCCACCACCCTACCACTCCCAC 1260

401 L S T V Q E D A A S L G P P P P Y H S H 420

1261 AAACACGTCTATCTTTCCAGACATCATTGCAAAGCTAAACCTCCCTCTAAAGACCCCGTC 1320

421 K H V Y L S R H H C K A K P P S K D P V 440

1321 ACACAACCCACTGCATATCCTCACAAGCATGGAATCGGTTATTTACCCCAGATTCCAATG 1380

441 T Q P T A Y P H K H G I G Y L P Q I P M 460

1381 CTGGAAGAGGAAGAAGCAGAGTTGGAGGACTACAGTGCTCACTGCTGCCGATGGATGGAC 1440

461 L E E E E A E L E D Y S A H C C R W M D 480

1441 TGCAGTGCAGTTTATGACCATAAGGAGGAGCTGGTGAGGCACATAGAGAAGCTACATGTG 1500

481 C S A V Y D H K E E L V R H I E K L H V 500

1501 GACCAAAGGAAGACGGAGGACTTCACCTGCTACTGGGTCGGCTGTCCACGCAACTTGAAG 1560

501 D Q R K T E D F T C Y W V G C P R N L K 520

1561 CCCTTTAATGCCCGATACAAGCTTCTCATCCACATGAGGGTCCATTCTGGAGAGAAGCCC 1620

521 P F N A R Y K L L I H M R V H S G E K P 540

1621 AACAAATGCTCGTTTGAAGGCTGCAAGAAGGCATTTTCTCGACTTGAAAACCTGAAGATC 1680　　　　exon4-5

541 N K C S F E G C K K A F S R L E N L K I 560

1681 CACCTGCGCAGTCACACGGGGGAGAAACCCTATCTGTGTCAGCACCCAGGATGCCACAAG 1740

561 H L R S H T G E K P Y L C Q H P G C H K 580

1741 GCTTTCAGCAACTCCAGTGACAGAGCCAAACACCAGCGCACACACCTGGACACAAAGCCG 1800　　　　exon5-6

581 A F S N S S D R A K H Q R T H L D T K P 600

1801 TACGCATGTCAGGTTCCTGGCTGTGCAAAGCGTTACACTGATCCCAGTTCATTAAGGAAA 1860

601 Y A C Q V P G C A K R Y T D P S S L R K 620

1861 CATTTGAAATCCCACTCTACAAGAGAGCGACAGTTACGCAAGAAGATGAAATCCTACACT 1920　　　　exon6-7

621 H L K S H S T R E R Q L R K K M K S Y T 640

1921 GATGGAAGTCTGGACACACTTACAGATTGTTTAACTATCCAACACCTTCAGCCAAACACT 1980

641 D G S L D T L T D C L T I Q H L Q P N T 660

1981 TCCCCTCTGGCAAGCCAAGACAACTTATCTCCTGGTGCCTCTCATGACTCATTCTCTGCT 2040　　　　exon7-8

661 S P L A S Q D N L S P G A S H D S F S A 680

2041 GCAGCACAAGGACACGACTCCTTCGGCGATCCTCACCTGACTCATTTGTTACCCATTCAA 2100

681 A A Q G H D S F G D P H L T H L L P I Q 700

2101 GATGGCCCCAGGTTTGCTGCACCTTGCCCTCATCAAGTGTTCTCTGAGGACTTGTGTGAG 2160　　　　exon8-9

701 D G P R F A A P C P H Q V F S E D L C E 720

2161 ACTTCCCCCTGCCTAACCACCAGTGGATCATTCCTTCAGAACAACTGTTATCTACAGCAG 2220

721 T S P C L T T S G S F L Q N N C Y L Q Q 740

2221 TTCAGCCAAGCTCCTGGTGTGGCAGATCACAGCCCAGGATTTGACGTCCACATTCAAGAG 2280　　　　exon9-10

741 F S Q A P G V A D H S P G F D V H I Q E 760

2281 GAAGAAATTACTGCTAATCGGGAGCTGCACAGCGCAGAGTACTACACTGTTTTGGACCAT 2340

761 E E I T A N R E L H S A E Y Y T V L D H 780

2341 GGCACAAATTAGGTTTTCTATCCAGTTGGATGAGAACCTTCACTGATGGATATTCTATTG 2400

781 G T N * 783

　　2401 CACTACTTTTTGCTACAGAGGGAGCACATCTTGTTTATGTTAGTTCAATGCCTCAAGCAG 2460

2461 ACAAAATGATGAAATAGTCAAAGATTCTATTTTTAAAAATAACTGTTAATGTGTTTGTTA 2520

2521 AAGCATCATAAATACATTATTCTTACTTCATAATTAATGTGCTAGCAGTTTTTTTTTCTT 2580

2581 TTTTCGTTTACTTGTTAGGTTGAGACGCAGTTCCTGTTCTGTATGTAGTACTTCAGGTCT 2640

2641 GATGGCATTTTGCACAATGTTAACACTGCAATCAAAGCAAAGTCTGCTATGGATCATGTT 2700

2701 TTTTTTCTCTTAGAGCCTTGTCTTTGTAACTATAGTAACAAGACACCCTCACCATTTCAA 2760

2761 TGTAATATTCCTGCATATCACCTGTTTCTTGAATAATGAAAGGACT 2806

Fig. S2
